# Usefulness of the HE4 biomarker as a second-line test in the assessment of suspicious ovarian tumors

**DOI:** 10.1007/s00404-013-2901-1

**Published:** 2013-05-31

**Authors:** Rafal Moszynski, Sebastian Szubert, Dariusz Szpurek, Slawomir Michalak, Joanna Krygowska, Stefan Sajdak

**Affiliations:** 1 Division of Gynecological Surgery, Poznan University of Medical Sciences, 33. Polna St., 60-535 Poznan, Poland; 2 Department of Neurochemistry and Neuropathology, Poznan University of Medical Sciences, Poznan, Poland; 3 Neuroimmunological Unit Polish Academy of Sciences, Poznan, Poland

**Keywords:** Suspicious adnexal mass, Biomarkers, HE4, Ovarian cancer, Subjective assessment, Ultrasonography

## Abstract

**Purpose:**

The aim of our study was the evaluation of HE4 usefulness as a test in assessment of ovarian tumors which are suspicious and difficult to classify correctly via subjective ultrasound examination.

**Methods:**

In this retrospective cohort study 253 women diagnosed with adnexal masses were examined preoperatively. Suspicious tumors (*n* = 145) were divided into groups of: “probably benign” (*n* = 70), “uncertain” (*n* = 34), and “probably malignant” (*n* = 41). “Uncertain” tumors were also assessed as “benign” (*n* = 11) or “malignant” (*n* = 23). The logistic regression model was performed to analyze if the serum marker improves the prediction of a malignant finding and net reclassification improvement (NRI) was calculated to measure diagnostic improvement.

**Results:**

Within the analyzed group 85 (58.6 %) benign and 60 (41.4 %) malignant tumors were confirmed histopathologically. The comparison of HE4 with subjective ultrasound assessment showed lowered NRI in the entire analyzed group as well as in the groups of tumors classified as “probably benign” or “probably malignant” (NRI = −0.16; *P* = 0.0139 and NRI = −0.133; *P* = 0.0489, respectively). The analysis of logistic regression model confirmed that biomarkers do not improve diagnostic accuracy. The difference between areas under ROC for HE4 (0.891) and CA125 (0.902) was not statistically significant (*P* = 0.760).

**Conclusions:**

After subjective ultrasound assessment, the addition of the second-line test—HE4 as well as CA125 serum level does not improve diagnostic performance. However, HE4 evaluation satisfies the clinical expectations of diagnostic tools for ovarian tumors and, thus, may be useful to less experienced sonographers.

## Introduction

Epithelial ovarian cancer (EOC) remains one of the most challenging problems in contemporary gynecological oncology worldwide. Its high incidence and the fact that it is especially an important cause of mortality among malignant diseases make the problems of EOC diagnosis and treatment very important. Research concerns the elaboration of effective screening programs and methods for early selection of women with ovarian cancer in preclinical stage [1]. It also focuses on helpful methods for preoperative malignancy prediction. If preoperative risk of malignancy is high, it is crucial to refer patients to gynecological oncology centers for surgical treatment, because the prognosis is better there [2].

Currently, transvaginal ultrasonography is the most effective method for prediction of malignancy. There are opinions that the subjective assessment of an experienced ultrasound examiner with a good quality ultrasound device can distinguish most benign and malignant ovarian tumors [3–6]. Mathematical models and morphological indices are also useful [7, 8]. Validation of the best mathematical models with comparison to the “pattern recognition” used by an expert sonographer confirms that the best diagnostic method is subjective assessment of the tumor, although the differences are not large [4]. But still a group of suspicious tumors which are difficult to classify correctly requires second-line tests [9, 10]. Improvement of this classification may be achieved by the assessment of biochemical markers. CA125 is commonly used, but is far from ideal [11]. The main drawbacks of CA125 are low sensitivity, specificity, and the risk of false-positive results in non-EOC malignant tumors as well as in benign conditions [12]. Furthermore, Valentin et al. [13] have shown that adding the CA125 measurement to ultrasonography assessment does not improve the accuracy of differential diagnosis of adnexal masses. In 2003, a novel serum biomarker HE4 was proposed as either a first- or a second-line screen for EOC and was registered for monitoring the disease status in women with ovarian cancer [1, 14]. HE4 is an 11–13kD protein that is a precursor to the human epididymal secretory protein E4. It is a member of the family of stable 4-disulfide core proteins [14]. Recently many markers were described in ovarian cancer diagnosis; however, in our opinion HE4 seems to be the most promising among them. HE4 in literature has been assessed as being a more specific marker than CA125 alone, and as very helpful in combination with CA125 for risk of malignancy prediction [15, 16].

The aim of our study was the evaluation of HE4’s usefulness as a test in assessment of ovarian tumors which are suspicious in a preoperative analysis by subjective ultrasound examination.

## Materials and methods

**Fig. 1:**
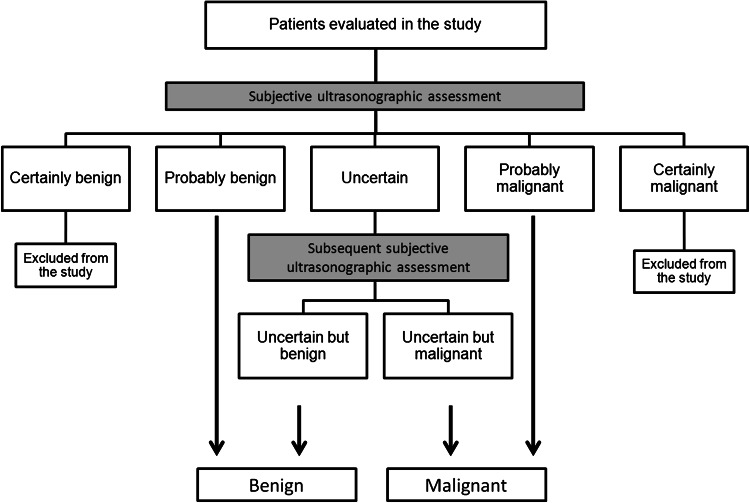
The diagnostic algorithm of subjective ultrasound classification of the risk of malignancy of analyzed tumors

In this retrospective cohort study 253 consecutive women diagnosed with adnexal masses were examined preoperatively with transvaginal ultrasonography by one experienced sonographist, between 2005 and 2011 in a tertiary gynecological oncology centre. The ultrasound examination was performed using an Aloka 3500 with a 7.5 MHz endovaginal probe and additionally with a transabdominal probe in large tumors. According to international ovarian tumor analysis (IOTA) guidelines, the examiner performed a subjective assessment of the risk of malignancy of any tumor [17]. Tumors were classified as “certainly benign” (*n* = 84), “probably benign” (*n* = 70), “uncertain” (*n* = 34), “probably malignant” (*n* = 41), and “certainly malignant” (*n* = 24). Tumors estimated to be “certainly benign” or “certainly malignant” were excluded, while the rest of the tumors were termed as “suspicious” tumors and included for further analysis. Subsequently, “suspicious” tumors were again classified as “benign” (*n* = 81) or “malignant” (*n* = 64) in a final subjective ultrasound assessment. Thus, the tumors classified as “uncertain” in the first evaluation, were secondly classified as “benign” or “malignant”. Whereas “probably benign” tumors were subsequently classified as “benign”, while “probably malignant” were regarded as “malignant”. The diagnostic algorithm of subjective ultrasound classification of the risk of malignancy of analyzed tumors is presented in Fig. 1. In general, the examiner judged unilocular and multilocular cysts without any papillary projection, even if smaller than 3 mm, or solid components to be benign. In some cases, specific diagnosis was possible (e.g., endometrioma, teratoma, etc.,) based on “pattern recognition” of the gray-scale ultrasound image and those tumors were classified as “certainly benign”. Cystic tumors with solid components and more complex, irregular tumors were judged to be malignant.

Tumors of borderline malignancy (Low Malignancy Potential, LMP) and metastatic adnexal tumors were classified as malignant adnexal masses.

Prior to operation, sera were collected for the determination of tumor markers HE4 and CA125 levels. HE4 serum levels were analyzed by EIA assay (Fujirebio Diagnostics AB Göteborg, Sweden). CA125 serum levels were assessed by immunoenzymatic test, ST AIA-PACK OVCA TOSOH Japan. We assessed the utility of CA125 in diagnosis of ovarian tumors with the use of two deferent cut-offs: the standard cut-off used in our hospital—35 IU/ml and the best cut-off calculated in the present study.

The usefulness of HE4 and CA125 assessment was estimated using the analysis of receiver-operating characteristics curves (ROC Curve). The ROC curve for subjective ultrasonographic assessment was calculated with the use of four levels of diagnostic confidence (“probably benign”, “uncertain, finally classified as benign”, “uncertain, finally classified as malignant” and “probably malignant”). ROC curves were constructed using MedCalc, version 10.4.0.0 computer software.

Net reclassification improvement (NRI), which assesses risk reclassification and is a measure of diagnostic improvement, was calculated based on the published formula [18]. There is a growing body of evidence that leads to recommendations for the application of NRI in research on new biomarkers introduced in clinical practice [19]. NRI enables the quantification of improvement of classification of events/non-events (e.g., “malignant”, “non-malignant” tumors) offered by new markers. Hence, the reclassification tables, which NRI focuses on, are constructed separately for subjects with and without events. Thus, NRI quantifies the correct movement in categories i.e., upwards for events and downwards for non-events. We calculated the NRI for each marker (HE4, CA125 at 35 and 95 IU/ml cut-off) as an addition to the subjective assessment of the entire analyzed group, and separately for tumors classified as “probably malignant” or “probably benign” and for “uncertain” tumors. In addition, NRI was used to determine the contribution of HE4 analysis to CA125 assessment. Furthermore, the logistic regression model including subjective ultrasound assessment with the addition of HE4 and CA125 was conducted.

Statistical analyses were conducted using Statistica for Windows ver. 6.1 (Statsoft, USA) PQStat ver 1.4.6 (PQStat Software, Poland). In the present study, the range was defined as a minimal and maximal value.

The study received ethics approval (05.2005) and all patients signed consent forms.

## Results

Within the analyzed group of 145 women 85 (58.6 %) benign and 60 (41.4 %) malignant tumors were confirmed histopathologically. Eleven LMP tumors were included in the malignant tumors group. The histological classification of benign and malignant tumors is presented in Table 1. Malignant tumors were classified according to the FIGO stage of the disease as follows: I stage, 19 patients; II stage, 6 patients; and III stage, 35 patients.

**Table 1:** Histopathological tumor characteristics

Tumor	Premenopausal	Postmenopausal	All patients	%
Benign				
Simple/functional/hemorrhagic cyst	12	4	16	18.8
Endometrioma	23	1	24	28.2
Teratoma	14	3	17	20.0
Serous cystadenoma	3	3	6	7.1
Mucinous cystadenoma	4	5	9	10.6
Tubo-ovarian abscess	4	1	5	5.9
Fibrothecoma/fibroadenoma/Brenner’s tumor	2	4	6	7.1
Leiomyoma	1	1	2	2.3
Total	63	22	85	100
Malignant				
Serous adenocarcinoma	6	19	25	41.7
Mucinous adenocarcinoma	0	2	2	3.3
Endometrioid adenocarcinoma	2	2	4	6.7
Clear cell adenocarcinoma	1	2	3	5.0
Undifferentiated carcinoma	6	6	12	20.0
Other	0	3	3	5.0
Serous cancer of LMP	3	0	3	5.0
Mucinous cancer of LMP	3	5	8	13.3
Total	21	39	60	100

The mean age of the patients studied was 47 years (range 15–84 years). The mean age was 41.7 years (range 15–74 years) and 54.6 years (range 21–84 years) for the patients with benign and malignant tumors, respectively. Eighty-four (57.9 %) patients were premenopausal, while 61 (42.1 %) patients were postmenopausal.

Median tumor volume was 497 cm^3^ (range 17–4,187 cm^3^) and 101 cm^3^ (range 13–4,017 cm^3^) in the malignant and benign tumor groups, respectively. The sonographic structure of the tumors analyzed is presented in Table 2.

**Table 2:** Sonographic structure of analyzed tumors

	Benign tumors *n* (%)	Malignant tumors *n* (%)	All tumors *n* (%)
Unilocular	13 (15.3)	2 (3.3)	15 (10.3)
Unilocular solid	18 (21.2)	4 (6.7)	22 (15.2)
Multilocular	20 (23.5)	5 (8.3)	25 (17.2)
Multilocular solid	25 (29.4)	36 (60)	61 (42.1)
Purely solid	7 (8.2)	13 (21.7)	20 (13.8)
Not classifiable	2 (2.4)	0 (0)	2 (1.4)
Total	85 (100)	60 (100)	145 (100)

The median serum concentration of HE4 in all benign tumors was 32.7 pmol/l (range 18.9–157.0) and in all malignant tumors was significantly (*P* < 0.001) higher, at a level of 183.5 pmol/l (range 19.3–4,246.7). If the subgroup of LMP tumors was separated, median HE4 concentrations in malignant and LMP tumors were significantly different (*P* < 0.001), 329.8 pmol/l (range 35.9–4,246.7) and 39.8 pmol/l (range 19.3–90.3), respectively. Whereas, the differences between HE4 concentrations in benign and LMP tumors were not statistically significant (*P* > 0.05).

The median serum concentration of CA125 in all malignant tumors was 650.4 IU/ml (range 9.0–3,657.0) and it was significantly (*P* < 0.001) higher than in all benign tumors, 21.1 IU/ml (range 4.2–525.1). In the subgroup of LMP tumors, the median for malignant and LMP tumor CA125 levels differed significantly (*P* < 0.01) (913.0 and 51.4 IU/ml, respectively). The difference in CA125 levels between benign and LMP tumors was not statistically significant (*P* > 0.05).

The results of HE4 and CA125 serum levels in the subgroups of patients according to FIGO classification are presented in Table 3.

**Table 3:** Median and range (minimum and maximum) of HE4 and CA125 serum levels according to FIGO classification

	FIGO I (*n* = 19)		FIGO II (*n* = 6)		FIGO III (*n* = 35)		*P* value
	Median	Range	Median	Range	Median	Range	
HE4 [pmol/l]	49.2	19.3–4,000	470.6	67.6–1,222.6	333.5	42.3–4,246.7	I vs. II *p* < 0.05 I vs. III *p* < 0.001 II vs. III *p* > 0.05
CA125 [IU/ml]	77.2	9.04–1,260.0	940.15	37.0–3,269.0	1,018.9	89.0–3,657.0	I vs. II *p* > 0.05 I vs. III *p* < 0.001 II vs. III *p* > 0.05

**Fig. 2:**
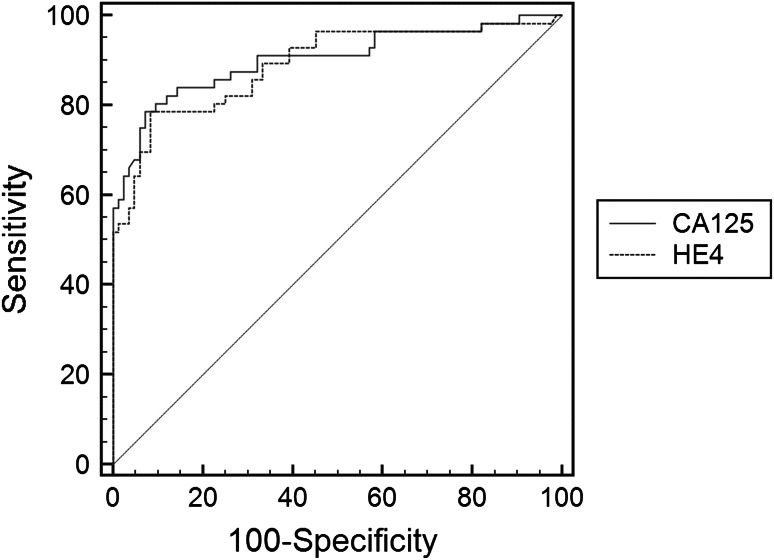
ROC curves for HE4 and CA125 among patients with ovarian tumors

The diagnostic usefulness of HE4 and CA125 serum level assessment as single tests was compared based on the area under ROC analysis (Fig. 2). The differences between areas under ROC for HE4 and CA125 were not statistically significant (*P* = 0.760). According to the ROC analysis, the best cut-off for HE4 serum level was set at 65 pmol/l. But, we realize that the sample size is very low to recommend general cut-offs. In our study the specificity (91.7 %), accuracy (86.9 %) and positive predictive value (87.3 %) of HE4 analysis were higher than in CA125 analysis, while sensitivity (80.0 %) was lower. Combination of HE4 and CA125, when either or both biomarkers’ serum concentrations were above the cut-off level was characterized by AU ROC = 0.866 (95 % CI 0.798–0.918). HE4 has higher diagnostic values than CA125, especially for its standard cut-off, 35 IU/ml. If the cut-off of CA125 is set at a level of 95 IU/ml in the analyzed group of “suspicious” tumors it gives better diagnostic performance almost as good as HE4.

The prognostic values of subjective ultrasonography assessment were very high, with sensitivity and specificity of 93.3 and 90.6 %, respectively. The area under ROC was 0.920 (95 % CI 0.863–0.958). Areas under ROC for CA125, 0.902 (95 % CI 0.840–0.946) and for HE4, 0.891 (95 % CI 0.827–0.937) were not as high as for subjective ultrasonography analysis, but the differences were not statistically significant (subjective assessment vs. HE4, *P* = 0.226; subjective assessment vs. CA125 *P* = 0.490). Similarly, the area under the ROC curve for the combination test composed of HE4 and CA125 (AU ROC = 0.866) was not significantly lower than for subjective assessment (*P* = 0.099). The prognostic values for subjective assessment, HE4 and CA125 levels are presented in Table 4, while, Table 5 summarizes the results of subjective ultrasonographic assessment.

**Table 4:** Comparison of prognostic values of subjective assessment, HE4 serum level, CA125 serum level in group of 145 “suspicious” ovarian tumors analyzed

Test	Sens	Spec	PPV	NPV	ACC	AU ROC
Subjective ultrasound assessment	93.3	90.6	87.5	95.1	91.7	0.923
HE4	80.0	91.7	87.3	86.7	86.9	0.891
CA125 (cut-off 35 IU/ml)	85.7	74.7	69.6	88.7	79.1	0.902
CA125 (cut-off 95 IU/ml)	78.6	91.7	86.3	86.5	86.4	0.902

*Sens* sensitivity, *Spec* specificity, *PPV* positive predictive value, *NPV* negative predictive value, *ACC* accuracy, *AU ROC* area under ROC

**Table 5:** The results of subjective ultrasonographic assessment in groups of malignant and benign tumors

Ultrasonographic classification	Histopathological diagnosis	
	Benign tumors	Malignant tumors
Probably benign (*n* = 70)	**67 (95.7** **%)**	3 (4.3 %)
Uncertain, finally classified as benign (*n* = 11)	**10 (90.9** **%)**	1 (9.1 %)
Uncertain, finally classified as malignant (*n* = 23)	6 (26.1 %)	**17 (73.9** **%)**
Probably malignant (*n* = 41)	2 (4.9 %)	**39 (95.1** **%)**

Bold values are statistically significant (*P* < 0.0001)

The comparison of HE4 and CA125 (at both analyzed cut-offs) with subjective ultrasound assessment showed lowered NRI across all analyzed groups as well as in the groups of tumors classified as “probably benign” or “probably malignant”. However, in the group of tumors classified as “uncertain”, the improvement was not statistically significant. The results are summarized in Table 6. NRI calculated for discrimination between malignant and benign tumors did not show significant improvement of the diagnosis when HE4 was compared with CA125 (NRI = −0.034, *P* = 0.973).

**Table 6:** Net reclassification improvement calculated for the assessment of second-line test benefits after subjective ultrasound evaluation

Marker	NRI	*P* value
Tumors classified as “probably benign”, “uncertain”, “probably malignant”		
HE4	−0.16	0.014
CA125 (cut-off = 35 IU/ml)	−0.319	0.001
CA125 (cut-off = 95 IU/ml)	−0.1765	0.018
Tumors classified as “probably benign” and “probably malignant”		
HE4	−0.133	0.049
CA125 (cut-off = 35 IU/ml)	−0.327	0.001
CA125 (cut-off = 95 IU/ml)	−0.234	0.006
Tumors classified as “uncertain”		
HE4	−0.042	0.817
CA125 (cut-off = 35 IU/ml)	0.201	0.209
CA125 (cut-off = 95 IU/ml)	0.07	0.676

The analysis of logistic regression including ultrasonographic assessment, CA125 and HE4 levels evaluation in discrimination between malignant and benign ovarian tumors, showed that only ultrasonography had significant impact on developing model. In that model, subjective ultrasonographic assessment correctly classified 93.3 % of cases. The analysis of logistic regression model confirmed that biomarkers do not improve diagnostic accuracy.

## Discussion

In the present study, we have found a lack of clinical utility of HE4 serum concentrations assessment in patients with ovarian tumors which are suspicious and difficult to classify correctly in subjective ultrasonography examination performed by an experienced examiner. Similar results were obtained for CA125. However, in the group of ovarian tumors included in the study, the HE4 assessment was a more specific and accurate test compared to CA125. The positive predictive value of HE4 estimation in our study exceeded the precision rate of CA125, especially for its standard cut-off.

Research concerned with “suspicious” ovarian tumors is especially important, because in this group the risk of false results in diagnostic tests is high. It is not very difficult to assess tumors at an advanced stage of the disease and then the decision to operate in an oncological center is obvious. Similarly, it is easy to diagnose a simple cyst or other tumors classified as “certainly benign”. In this group, the risk of malignancy is extremely low. This is why the most important and interesting tumors are those which pose problems in ultrasound evaluation. According to the IOTA group’s recent publication by Valentin et al. [20], only 7–10 % of masses are suspicious and difficult to classify. This concerns tumors which are completely “uncertain” as whether they are malignant or benign in sonographic assessment. In our research this was 13.4 % (34/253) of tumors, but for the final analysis of “suspicious” masses we also included tumors which were “probably malignant” and “probably benign”, where the decision is in some way uncertain as well. This is why the group of analyzed tumors consisted of 57 % (145/253) of all patients diagnosed with adnexal masses.

Valentin et al. [10] suggest that logistic regression models do not solve diagnostic problems in suspicious pelvic masses. Daemen et al. [9] suggest that in the group of tumors where an examiner is wholly uncertain about the diagnosis, new tests may well help in better classification of these patients. For this reason, we conducted the study looking at the role of a novel ovarian cancer marker as a second-line test.

Hellström et al. [14] investigated the HE4 protein in an ELISA assay in ovarian cancer patients, benign ovarian diseases, and health checks in 2003. They concluded that HE4 is overexpressed in ovarian cancer patients and has comparable sensitivity and specificity to CA125. The advantage of HE4 as a biomarker was the better detection of early cases of ovarian cancer where at 95 % specificity, sensitivity for HE4 and CA125 were 86 and 71 %, respectively. In our analysis for all FIGO stages for 95 % specificity, sensitivity for HE4 and CA125 were 65.0 and 76.8 %, respectively.

Moore et al. [16] proposed a predictive model for calculation of risk of ovarian cancer based on the combination of HE4 and CA125 serum levels. This multicenter, prospective study confirmed the clinical usefulness of the proposed model for the entire group of patients and also subgroups of premenopausal and, especially, postmenopausal women. The Risk of Ovarian Malignancy Algorithm (ROMA) has been tested in several clinical trials [15, 16]. Van Gorp et al. [21] performed a prospective validation of ROMA, HE4 and CA125 assessments and concluded that neither of these tests are better than CA125. Contrary considerations were presented by Molina et al. [15] who noticed that HE4 is more specific than CA125 and has at least the same sensitivity as CA125. In that paper, Molina et al. [15] also noted that the level of false-positive CA125 results is high, especially in premenopausal women. In another paper presented by Van Gorp et al. [22], the authors suggest that subjective ultrasound assessment has the highest area under the ROC = 0.968, and is better than the risk of malignancy index (0.931) and ROMA (0.893) both in pre- and in postmenopausal women. Those authors concluded that subjective assessment by ultrasound remains superior in discriminating malignant from benign ovarian masses. Our data are similar with these findings, where AU ROC for subjective ultrasound was 0.923 and for combination of HE4 and CA125, 0.866 (*P* = 0.099).

According to the results presented in our study, we conclude that subjective assessment was the best, as far as the current state of the art for all tests under comparison is concerned. Therefore, the training of ultrasound specialists, using the most experienced persons to scan patients preoperatively, is the main prerequisite for the maintaining the largest available accuracy in the diagnostics and quality of scanning of adnexal masses. This opinion is also presented by Timmerman et al. [3]. Also Franchi et al. [23] report, in a multicenter prospective study of 174 women with adnexal masses, that ultrasound expertise remains superior in discriminating malignant masses in comparison with the ROMA algorithm, CA125 or HE4 alone. They also report that HE4 has the highest specificity 92 %, better than ROMA and CA125, 83.8 and 66.7 %, respectively.

This is, however, limited by the subjective assessment in ultrasound examination, which is effective only when it is performed by experienced clinician. Biomarker analysis is more readily available to less experienced centers. HE4 alone or in combination with CA125 should, therefore, be used as an adjunct to less experienced sonographers. Franchi et al. [23] also present the opinion that a combination of biomarkers could offer an aid to less experienced sonographers in the preoperative triage of adnexal masses.

We have shown, that in all analyzed tumors and, especially in the “probably benign” and “probably malignant” tumor groups, subjective ultrasound assessment has such high prognostic values that adding biomarker evaluation worsens diagnostic performance. In the group of “uncertain” tumors, subjective assessment and biomarker evaluation both have low sensitivity and specificity, and in this situation a search for new diagnostic tests is necessary.

In conclusion, our study confirms the significance of subjective ultrasound assessment as the best single diagnostic test in malignancy prediction. After first-line subjective evaluation with ultrasonography, a portion of tumors still remains suspicious. In this situation, HE4 and CA125 serum levels do not improve the diagnostic accuracy. However, HE4 evaluation satisfies clinical expectations for a test to be a diagnostic tool in assessing ovarian tumors. The HE4 serum level has higher specificity, accuracy and positive predictive value than CA125 especially at its standard cut-off. A higher cut-off for CA125 in the group of “suspicious” ovarian tumors should be considered. These two biomarkers are complementary and may be useful for less experienced sonographers. In some situations, assessment using all possible methods is still not enough to identify the character of the disease and to exclude malignancy. So far, none of the analyzed biochemical tests has proved suitable as a second-line test in tumors where subjective evaluation yielded an uncertain result.
